# Examining the influence of environmental factors on *Acanthamoeba castellanii* and *Pseudomonas aeruginosa* in co-culture

**DOI:** 10.1371/journal.pone.0305973

**Published:** 2024-06-24

**Authors:** Rhiannon E. Cecil, Deborah R. Yoder-Himes

**Affiliations:** Biology Department, University of Louisville, Louisville, Kentucky, United States of America; Nova Southeastern University, UNITED STATES

## Abstract

Exploration of interspecies interactions between microorganisms can have taxonomic, ecological, evolutionary, or medical applications. To better explore interactions between microorganisms it is important to establish the ideal conditions that ensure survival of all species involved. In this study, we sought to identify the ideal biotic and abiotic factors that would result in high co-culture viability of two interkingdom species, *Pseudomonas aeruginosa* and *Acanthamoeba castellanii*, two soil dwelling microbes. There have been limited studies showing long-term interactions between these two organisms as co-culture can result in high mortality for one or both organisms suggesting a predator-predator interaction may exist between them. In this study, we identified biotic and abiotic conditions that resulted in a high viability for both organisms in long-term co-culture, including optimizing temperature, nutrient concentration, choice of bacterial strains, and the initial ratio of interacting partners. These two species represent ideal partners for studying microbial interactions because amoebae act similarly to mammalian immune cells in many respects, and this can allow researchers to study host-pathogen interactions *in vitro*. Therefore, long-term interaction studies between these microbes might reveal the evolutionary steps that occur in bacteria when subjected to intense predation, like what occurs when pathogens enter the human body. The culture conditions characterized here resulted in high viability for both organisms for at least 14-days in co-culture suggesting that long-term experimental studies between these species can be achieved using these culture conditions.

## Introduction

*Pseudomonas aeruginosa* is an opportunistic human pathogen that is commonly isolated from other natural environments such as soil or freshwater bodies of water. It is a leading cause for nosocomial infections in the U.S. and can infect nearly every organ system in the mammalian body including the skeletal system, cardiovascular system, digestive system, integumentary system, reproductive system, and respiratory system [1–6]. Its ability to survive in such a wide range of habitats is largely due to its large, plastic genome leading to wide metabolic capabilities [7, 8]. *P*. *aeruginosa* is also known to reduce the size of its genome via gene deletions to become highly adapted to a specific niche within a human host [9, 10]. *P*. *aeruginosa* has a wide arsenal of virulence factors which gives it a competitive edge over both prokaryotic and eukaryotic competitors within natural and anthropogenic environments [11–24].

*Acanthamoeba castellanii* is a natural predator of *P*. *aeruginosA. A. castellanii* is a free-living, single-celled, lobose amoeba species and is a model organism to study phagocytosis [25, 26]. Amoebae and phagocytic human immune cells, such as macrophage, share many conserved proteins and traits such as P_21_-activated kinase, Ras and Rab proteins, PI phosphatases and P1 kinases, mannose binding proteins, and phagocytosis [27–30]. Due to these similarities, it has been proposed that adaptations acquired by previously innocuous bacteria to escape/kill amoebae can, incidentally, result in virulence factors effective against human immune cells. One example organism for studying host-pathogen interactions between eukaryotes and facultative bacterial pathogens is *Legionella pneumophila*, which is an aquatic bacterium that survives and replicates intracellularly in eukaryotic cells. Amoebae are its natural host but, upon infecting humans, this organism is able to survive inside monocytes and macrophages using the same mechanisms of entry and intracellular survival strategies employed for their intracellular lifestyle within amoebae [31–33]. *A. castellanii* itself is also an opportunistic human pathogen with infections resulting in ocular keratitis and can also cause a rare form of encephalitis called *Acanthamoeba* granulomatous encephalitis [30]. These amoebae are found in both natural and anthropogenic water sources such as ponds or sink drains respectively. *A. castellanii* exists in two forms: a metabolically active, phagotrophic trophozoite form and a cryptobiotic cyst form. Its lifecycle involves a growth phase and two cellular differentiation stages: encystment and excystment [34]. In the metabolically active stage of the amoeba’s life cycle, the cell is referred to as a trophozoite. Trophozoites are motile, reproduce mitotically, and can phagocytose prey [35, 36]. Because *A. castellanii* is a proven predator of *P*. *aeruginosa* [37, 38] and can be found in the same types of environmental and man-made reservoirs, it would make for an ideal natural host to examine host-pathogen interactions.

One limitation to studying host-pathogen interactions using bacteria and amoeba is that often the interacting partners kill each other quite readily under certain abiotic and biotic conditions [37, 39–41]. This suggests that predator-predator interactions may also occur between these species rather than a strictly predator-prey interaction. Therefore, identifying conditions that maximize both the survival and the interaction between the partners is critical for understanding the molecular changes that are involved in the host-pathogen interactions and that may change over time.

In this study, we sought to optimize conditions to allow survival of *P*. *aeruginosa* and *A. castellanii* in co-culture for short- or long-term interaction experiments. We examined the effect of abiotic factors (temperature and concentration of nutrients) and biotic factors (bacterial isolate life history and concentration of interacting organisms) to maximize survival of both organisms in long-term co-culture. With the proper conditions identified, ecological and evolutionary interactions between these microorganisms can be further explored at the molecular and organismal level.

## Materials and methods

### Strains used in this study, culturing, and cell maintenance

Strains used in this study are described in Table 1. *A. castellanii* strain 30010 was obtained from the ATCC. Clinical *P*. *aeruginosa* strains were obtained from Norton’s Children’s Hospital microbiology lab (strains B84725, B80398, B80427, B80422, B80425, B84723, and PA3) from cystic fibrosis sputum. Environmental *P*. *aeruginosa* strains were isolated from household sink drains in Louisville, Kentucky, U.S.A. (SRP3151, SRP 17–047, and SRP 17–055) [42]. *A. castellanii* cells were maintained routinely in 15 mL of HL5 (per L: 0.5 g KH_4_PO_4_; 0.5 g Na_4_HPO_4_; 7 g yeast extract; 14 g proteose peptone; purchased premixed from Formedium, Catalog #HLG0102) [25] in T-75 tissue culture flasks at room temperature (22°C) until they had reached confluence (∼ 5 days). The cells were passaged by using a cell scraper to dislodge the trophozoites, then a sterile Pasteur pipet with suction was used to remove the culture from the flask. One mL of the culture was added back to the flask along with 14 mL of fresh HL5. *P*. *aeruginosa* cells were maintained in LB Lennox broth (per L: 10 g tryptone, 5 g yeast extract, 5 g NaCl; premixed from Hardy Diagnostics, Catalog #C7653) with aeration or on LB Lennox agar plates (IBI Scientific, Catalog #IB49121) prepared according to the manufacturer’s instructions and incubated at 37°C unless otherwise noted.

**Table 1:** *Pseudomonas aeruginosa* strains used in this experiment and their respective phenotypes.

Strain	Origin	Colony Phenotype	References
PA B80398	Clinical–CF sputum sample	Normal	This study
PA B80427	Clinical–CF sputum sample	Small colony	This study
PA B84725	Clinical–CF sputum sample	Mucoid	This study
PA B84723	Clinical–CF sputum sample	Normal	This study
PA B80422	Clinical–CF sputum sample	Small colony	This study
PA B80425	Clinical–CF sputum sample	Small colony	This study
PA 3	Clinical–CF sputum sample	Normal	This study
SRP 3151	Environmental–bathroom sink drain	Normal	[42, 43, 44]
SRP 17–047	Environmental–bathroom sink drain	Normal	[42, 43, 44]
SRP 17–055	Environmental–kitchen sink drain	Normal	[42, 43, 44]

### General method for co-culture experiments

*A. castellanii* was cultured in 100% HL5 at room temperature in T-75 tissue culture flasks until confluent. Cells were scraped with a tissue culture scraper to dislodge cells from the surface and counted on a hemocytometer. Approximately 6x10^3^ cells of *A. castellanii* based on hemocytometer estimates, were added to 4 mL of fresh 100% HL5 in 6-well tissue culture-treated polystyrene plates. The cells were allowed to incubate in the HL5 for 30 minutes at room temperature to allow the cells to anneal to the well bottom. The HL5 was removed via a Pasteur pipet with vacuum suction and the cells were washed 2 times with 4 mL sterile PBS. Then 8 mL of fresh sterile indicated medium was added to each well. *A. castellanii* cells were cultured in monoculture or in co-culture with *P*. *aeruginosa* strains, grown to mid-log phase in 5 mL LB Lennox broth, under the conditions listed below. *A. castellanii* and *P*. *aeruginosa* cells were enumerated on the indicated days as described below.

### Co-Culture variable optimization

Strain optimization: Ten total *P*. *aeruginosa* strains were selected and are listed in Table 1. Each was cultured in LB Lennox broth and added to *A. castellanii* as indicated above. Co-cultures and mono-cultures were diluted into 8 mL sterile PBS in 6-well tissue culture plates for the duration of the interaction study to force bacterial-amoeabal interaction. Quantitatively, the ratios were set-up to reflect a 1:1 ratio of bacteria:amoeba for each strain combination. Cultures were allowed to incubate over 7 days at 22°C and were enumerated at 3, 5, and 7 days post-inoculation.

Impact of growth medium concentration: *A. castellanii* and *P*. *aeruginosa* strains were cultured in monoculture or in co-culture in 6-well tissue culture plates containing 8 mL of 0.1% HL5, 1% HL5, 10% HL5, or 100% HL5 at 22°C for 14 days. Each strain was tested with biological triplicates. The cells were cultured in a 10 *A. castellanii* to 1 *P*. *aeruginosa* ratio. Every two days the supernatant was removed and replaced with 8 mL of the respective medium. *A. castellanii* and *P*. *aeruginosa* cells were enumerated on days 1, 7, and 14.

Impact of temperature: *A. castellanii* and *P*. *aeruginosa* strains were cultured in a 10 *A. castellanii* to 1 *P*. *aeruginosa* ratio in 8 mL of 1% HL5 in 6-well tissue culture plates as described for the medium concentration experiment above except the cultures were incubated at either 22°C, 30°C, or 37°C in 1% HL5 medium for 14 days.

Impact of starting ratio of *A. castellanii* and *P*. *aeruginosa*: *A. castellanii* and *P*. *aeruginosa* were cultured in a 10 *A. castellanii* to 1 *P*. *aeruginosa* ratio as described for the medium concentration experiment above except the cultures were mixed at MOIs of 100:1, 10:1, 1:1, 1:10, or 1:100 amoebae:bacteria and cultures were grown at 22°C in 1% HL5 medium for 14 days. `

### Enumeration of *A. castellanii* and *P*. *aeruginosa*

*A. castellanii* cells were enumerated via hemocytometer prior to the initiation of each experiment. *A. castellanii* was also subsequently enumerated via bright field microscopy at 400X magnification by examining three replicate images within each well to assess the number of surviving trophozoites and cysts present during and at the end of each experiment. *P*. *aeruginosa* cells were enumerated via serial dilution and plating on Lennox agar. Cysts were distinguished from trophozoites based on cellular morphology. Trophozoite appear as lobose amoebe cells with visible pseudopodia [45]. Cysts, on the other hand, appear as smooth circular cells with two distinct visible layers referred to as the endocyst and the exocyst [30, 46].

### Statistics

GraphPad Prism v5.04 was used for data analysis. Data were analyzed with either t-tests, one-way ANOVAs with either Dunnett’s or Tukey’s post-test, or two-way ANOVAs as indicated in each analysis. The effects of life history could not be directly analyzed in Figs 3–5 due to lack of statistical power as only one environmental strain was tested in those experiments. When required, values of -1, +0, or +1 of the limits of detection (either above or below) were substituted for statistical purposes only.

## Results

To better understand the relational dynamics between *P*. *aeruginosa* and *A. castellanii* in co-culture, we optimized several variables to ensure long-term survival of both partners. We also sought to understand how life history (clinical versus environmental) of the bacterial strains impacted the relationship with the amoebae. Further, we sought to optimize abiotic factors (medium and temperature) of the cultures, and biotic factors (ratio of bacteria to amoebae) in order to extend co-culture survival of both organisms. The contribution of each partner to survival was also explored as *P*. *aeruginosa* might serve as a food source for the amoebae [47, 48] though at least some strains of *P*. *aeruginosa* may have adaptations that allow it to survive phagolysosome-mediated destruction following engulfment [49–51] In contrast, amoebae excretions/secretions or the amoebae themselves can serve as a food source for *P*. *aeruginosa* in co-culture [39, 41, 52–54].

### Impact of *P*. *aeruginosa* strain on *A. castellanii* survival

The ecological niche from which the *P*. *aeruginosa* strains originated may play a strong role in their interaction with *A. castellanii* as different phenotypes of *P*. *aeruginosa* display different virulence characteristics. For example, mucoid colony phenotypes or small colony variants of *P*. *aeruginosa* are associated with chronic infections and lower virulence factor expression while normal phenotypes are associated with acute infections and higher virulence

factor expression [55–57]. Ten different *P*. *aeruginosa* strains (Table 1) were tested for their effects on co-culture survival dynamics with *A. castellanii* over 7 days to determine if any of the *P*. *aeruginosa* strains were either killed by *A. castellanii* or conversely killed *A. castellanii*. Further, we determined whether *A. castellanii* cells remained in their actively growing state (trophozoite) or encysted in the presence of these strains.

**Fig 1:**
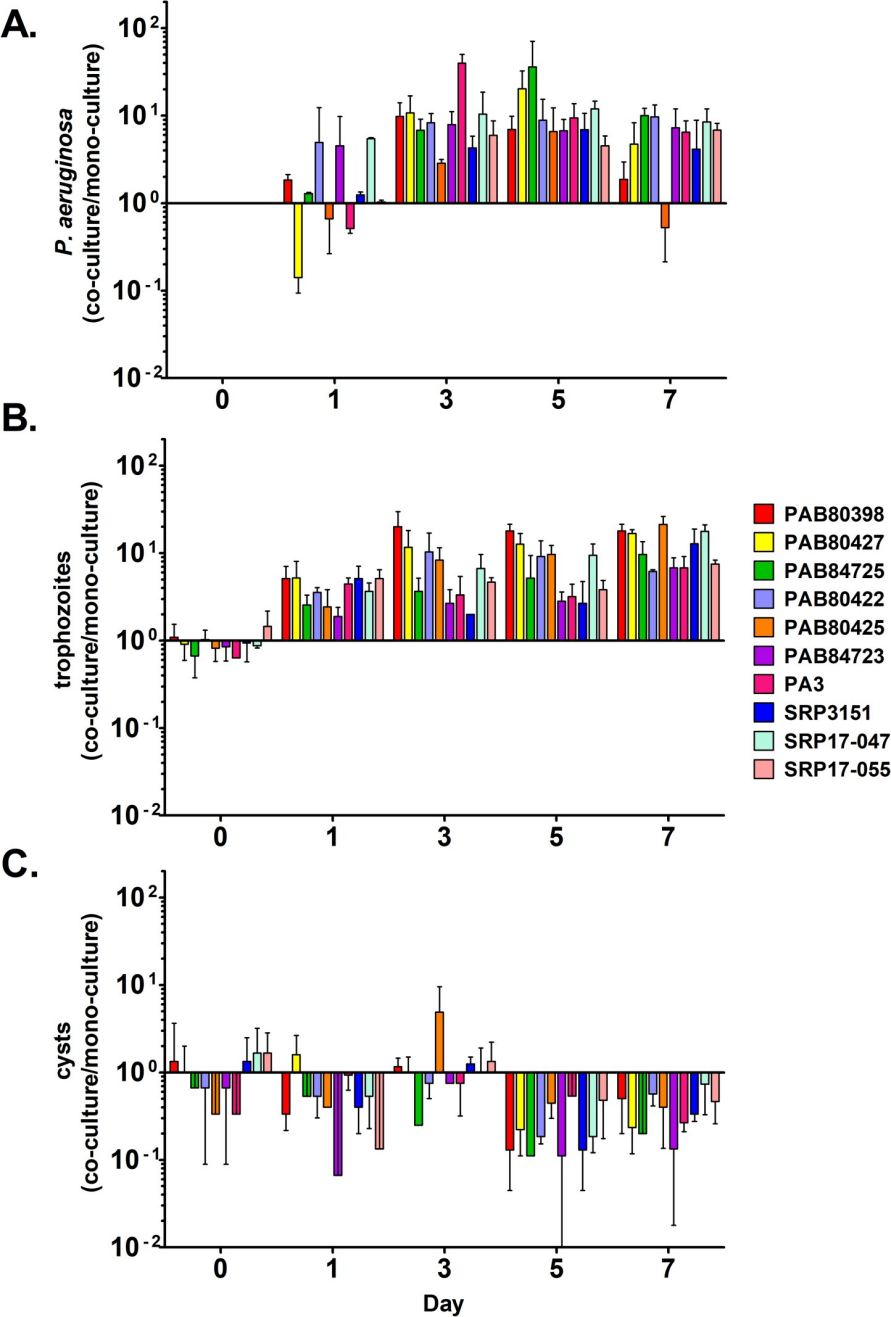
*A. castellanii* exhibits increased trophozoite survival and decreased encystment in co-culture with *P*. *aeruginosa* compared to monoculture. In all panels, 10^0^ represents the number of cells or CFUs present in monoculture at each time point. (A) Ratio of *P*. *aeruginosa* concentrations in co-culture with *A. castellanii* to *P*. *aeruginosa* survival in monoculture. (B) Ratio of the number of *A. castellanii* trophozoites present in co-culture with each *P*. *aeruginosa* strain compared to monoculture. (C) Number of *A. castellanii* cysts present in co-culture with *P*. *aeruginosa* compared to monoculture. Data analyzed with two-way ANOVA (S1 Table). Error bars represent standard deviations of the mean.

All *P*. *aeruginosa* strains were able to survive in the presence of *A. castellanii* over the course of the experiment (Fig 1A). After only 24 hours, several of the clinical strains, including B80427, B80425, and PA3, showed a relatively small reduction in survival compared to all of the strains grown in the absence of amoebae; however, all of them rebounded by day 3 or 5. Interestingly, PA B80425 did not remain at elevated levels on day 7 and instead showed reduction in growth compared to mono-culture suggesting that it is less robust than the other strains when in the presence of amoebae. The environmental isolates all survived at a greater concentrations in co-culture than in mono-culture over all 4 time points suggesting that these particular strains, at least, were well-prepared for co-culture with amoebae, which is not surprising as both *P*. *aeruginosa* and *A. castellanii* are likely to share environmental niches and *A. castellanii* has been shown to aid in the extracellular survival of other bacterial species [58, 59]. An analysis of the *P*. *aeruginosa* concentrations in monoculture and co-culture showed that bacterial concentrations typically peaked after day 3 and remained high out to 7 days but were only generally increase 1–2 logs (S1 Fig). For two of the strains, the bacterial conditions decreased slightly (<1-log) after 7 days but most strains retained their peak values at this time point. Bacterial concentrations were typically higher in the co-culture conditions compared to the monoculture conditions as well.

*A. castellanii* survived and stayed in trophozoite form when in co-culture with all 10 *P*. *aeruginosa* strains at days 1, 3, 5, and 7 compared to monoculture (Fig 1B), perhaps owing to the food source of *P*. *aeruginosa* in these cultures. *A. castellanii* trophozoite numbers were generally highest when co-cultured with PA B80389 (normal colony, clinical isolate), PA B80427 (small colony, clinical isolate), PA B80425 (normal colony, clinical isolate), and SRP 17–047 (normal colony, environmental isolate) (Fig 1B). Raw *A. castellanii* trophozoite and cyst counts are provided in S2 Fig. Data tables for 2-way ANOVA results are available in S1 Table.

Encystment generally decreased over the course of the experiment in co-culture compared to monoculture though this effect was variable depending on the *P*. *aeruginosa* strain with which *A. castellanii* was co-cultured (Fig 1C). This variability is most likely due to the low numbers of cysts overall in most conditions except in the monoculture in which encystment occurred to a much higher concentration than any of the co-culture conditions (S2 Fig). We expect that the decrease in encystment specifically in co-culture over time comes from competition with *P*. *aeruginosa* for nutrients as the overall trophozoite and cyst concentrations in co-culture remain steady after ∼1–5 days.

From these experiments, it appears that *A. castellanii* was able to survive with all 10 *P*. *aeruginosa* strains tested, at least for the 7-day duration of the experiment. Trophozoite survival was greater in co-culture with *P*. *aeruginosa* than in monoculture indicating that *A. castellanii* is able to readily predate on all of these *P*. *aeruginosa* isolates. We also concluded that *P*. *aeruginosa* grew more robustly in co-culture with *A. castellanii* than in monoculture, which suggests that the amoebae are either serving as a food source themselves or are producing products that can be metabolized by *P*. *aeruginosa*.

To assess whether life history of the bacterial isolates is important for amoebae survival, we grouped the strains, those from humans and those from sink drains, and tested for significant differences in the number of surviving trophozoites. On average, *A. castellanii* trophozoites were more abundant when co-cultured with clinical *P*. *aeruginosa* strains than when cultured with environmental strains (S3 Fig). These results were not significantly different, suggesting that origin of isolation does not appear to influence these interactions. However, we should note that these drain isolates may have recently been associated with human hosts as they were isolated from households, and this could mean that they are more human-adapted rather than true environmental (i.e. non-human associated).

Four of the ten *P*. *aeruginosa* strains tested were chosen for further testing. Three strains were of clinical origin (B80398, B84725, and B80427) and represent three major colony phenotypes (normal, mucoid, and small colony variant respectively). The other strain was of environmental origin, SRP3151 (normal phenotype). The three clinical isolates were chosen based on their colony phenotypes and the environmental isolate. SRP 3151 was chosen due to its use in other studies [42, 43, 44] and because its genome has been sequenced and in case further transcriptional or metabolic exploration is required in the future.

### Influence of nutrient concentration

Nutrient levels could strongly influence the interactions between amoebae and bacteria. *A. castellanii* grows axenically in nutrient rich media, such as HL5, but can also grow in nutrient poor media using bacteria as a food source. To elucidate the trophozoite growth patterns and encystment rates of *A. castellanii* in different nutrient concentrations in the absence of bacteria, *A. castellannii* was cultured in either 1X PBS, 100% HL5 or two intermediate media (80%/20% of each media) over the course of 6 days. Trophozoite levels remained high over the course of the 6 days in media with 80% or 100% HL5 but decreased steadily over the 6 days in 20% HL5 or 1X PBS (S4 Fig). In contrast, encystment levels rose initially for all media conditions for the first two days and then held steady at low nutrient concentrations (20% HL5 and 1X PBS) but continued to rise in higher nutrient concentrations. Trophozoite and cyst patterns were further explored in mono-culture at a finer scale of different concentrations of HL5 with the general trend that the higher the medium concentration, the greater the trophozoite levels and the greater the encystment levels, presumably because there just more trophozoites in the culture that encysted. The concentration of trophozoites tended to peak at days 0–2 and remained steady thereafter.

To better understand how nutrient levels influenced co-culture survival for *P*. *aeruginosa* and *A. castellanii*, *A. castellanii* cells were cultured in monoculture or in co-culture with *P*. *aeruginosa* in 0.1% HL5, 1% HL5, 10% HL5, and 100% HL5 for up to 14 days. We hypothesized that the low nutrient conditions (0.1% or 1% HL5) would promote survival of *A. castellanii* in co-culture with *P*. *aeruginosa* as co-culturing these organisms in high nutrient medium results in high *A. castellanii* mortality based on pilot experiments.

**Fig 2:**
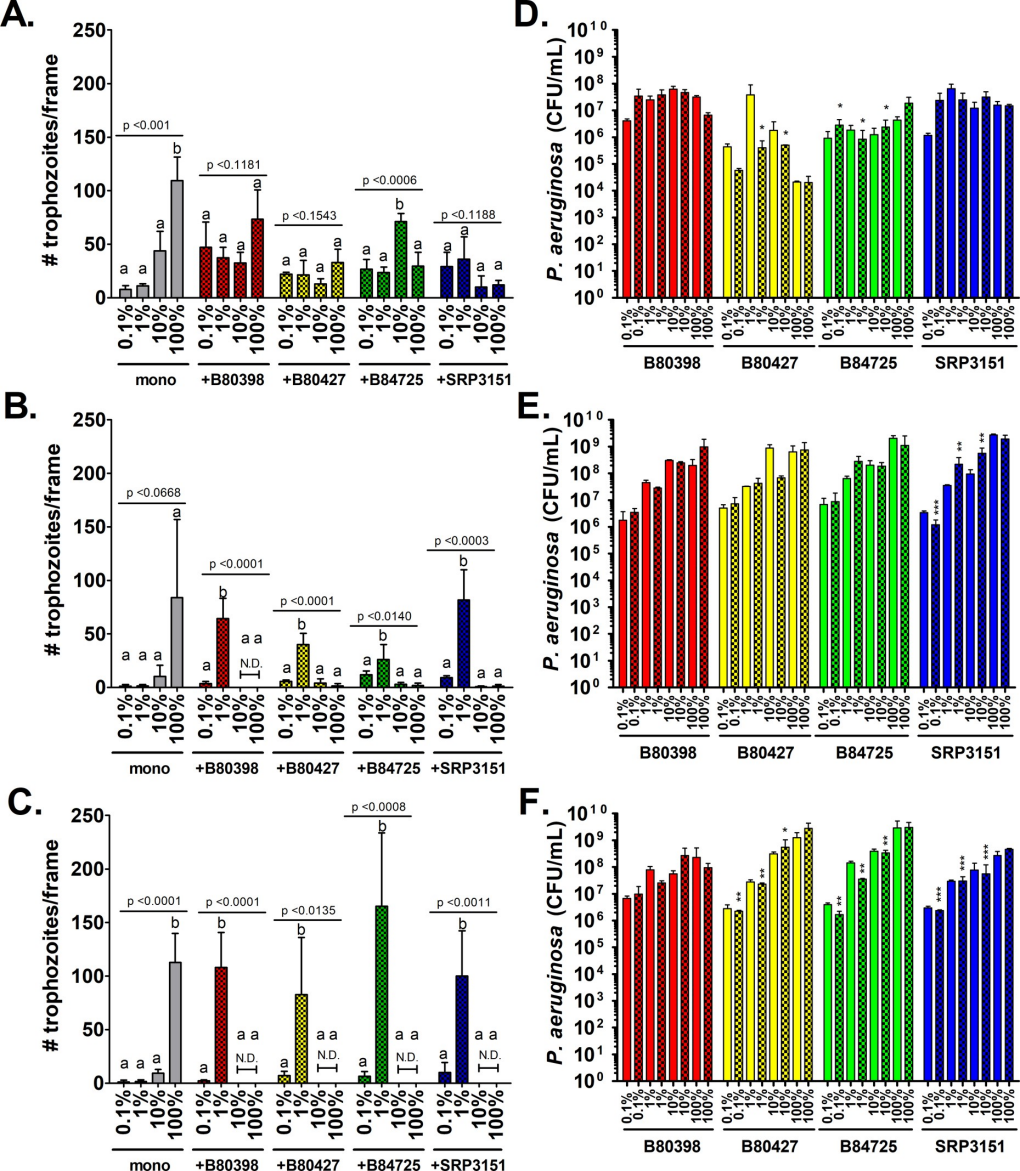
*A. castellanii* trophozoite survival in co-culture with *P*. *aeruginosa* is maximized when cultured in 1% HL5. *A. castellanii* was cultured in monoculture or in co-culture with four *P*. *aeruginosa* strains in 0.1% HL5, 1% HL5, 10% HL5 and 100% HL5 at 22°C. *P*. *aeruginosa* cells and *A. castellanii* trophozoites were enumerated after (A & D) 1 day, (B & E) 7 days, and (C & F) 14 days. For all panels, solid bars indicate concentrations in monoculture and checked bars indicate concentrations in co-culture. Error bars represent standard deviation of the mean. Data were analyzed with one-way ANOVA with Tukey’s post-test (n = 3 biological replicates). Lowercase letters indicate individual comparisons within each strain (but not between strains) and overall p-values for each group are indicated for each independent group comparison.

In general, *A. castellanii* trophozoites in monoculture were significantly more abundant when cultured in 100% HL5 compared to the all of the lower concentrations of HL5 tested (Fig 2A–2C) at most time points. However, amoebae trophozoite numbers co-cultured with 3 of the 4 *P*. *aeruginosa* strains were not significantly different between the nutrient concentration groups after one day (Fig 2A). In contrast, *A. castellanii* trophozoites were significantly more abundant when co-cultured in 1% HL5 than in 0.1%, 10%, or 100% HL5 at days 7 and 14 (Fig 2B and 2C). *A. castellanii* cyst concentrations had a trend of being higher in at 1- and 7-days post-inoculation in mono- and co-culture but by day 14, *A. castellanii* cysts were not found in 10% and 100% HL5 (S5 Fig). Tied together with the trophozoite data, it appears that in higher concentrations of nutrients, *A. castellanii* tends to die between 7- and 14-days post-inoculation as there are few trophozoites and few cysts.

In contrast, *P*. *aeruginosa* survived at high concentrations (∼>10^5^ CFU/mL) in monoculture and in co-culture with *A. castellanii* in all nutrient concentrations tested (Fig 2D–2F). However, *P*. *aeruginosa* CFU/mL tended to be highest when cultured in 100% HL5 compared to the other concentrations tested particularly at days 7 and 14 (Fig 2E–2F). Taken together, we can conclude that 1% HL5 supports the co-culture of *A. castellanii* with *P*. *aeruginosa* and while higher nutrients concentrations support *P*. *aeruginosa* for long-duration experiments; however, this concentration of HL5 does not support the growth/survival of *A. castellanii* in monoculture. Therefore, we chose to use 1% HL5 for the remaining experiments as it most robustly supported *A. castellanii*, which was much more sensitive to nutrient concentrations than *P*. *aeruginosa*.

### Effect of temperature on co-culture population dynamics

Temperature is another abiotic factor that could have an immense effect on the co-culture survival of one or both of the studies organisms. It has been shown that *P*. *aeruginosa* alters its virulence factor expression depending on culture temperatures [60, 61]; therefore, it is imperative to determine how temperature influences the interactions between these pathogens. We hypothesized that, of the temperatures tested, room temperature (22°C) would be the best growth temperature for survival of both partners when co-culturing these two organisms as 22°C is roughly the temperature these organisms would experience in sink drains. The optimal growth temperature for *A. castellanii* ranges from 22°C to 32°C *in vitro* [62]. *P*. *aeruginosa* is able to survive/grow at a temperature range from 4°C to 42°C, with 37°C being its optimal growth temperature [60].

**Fig 3:**
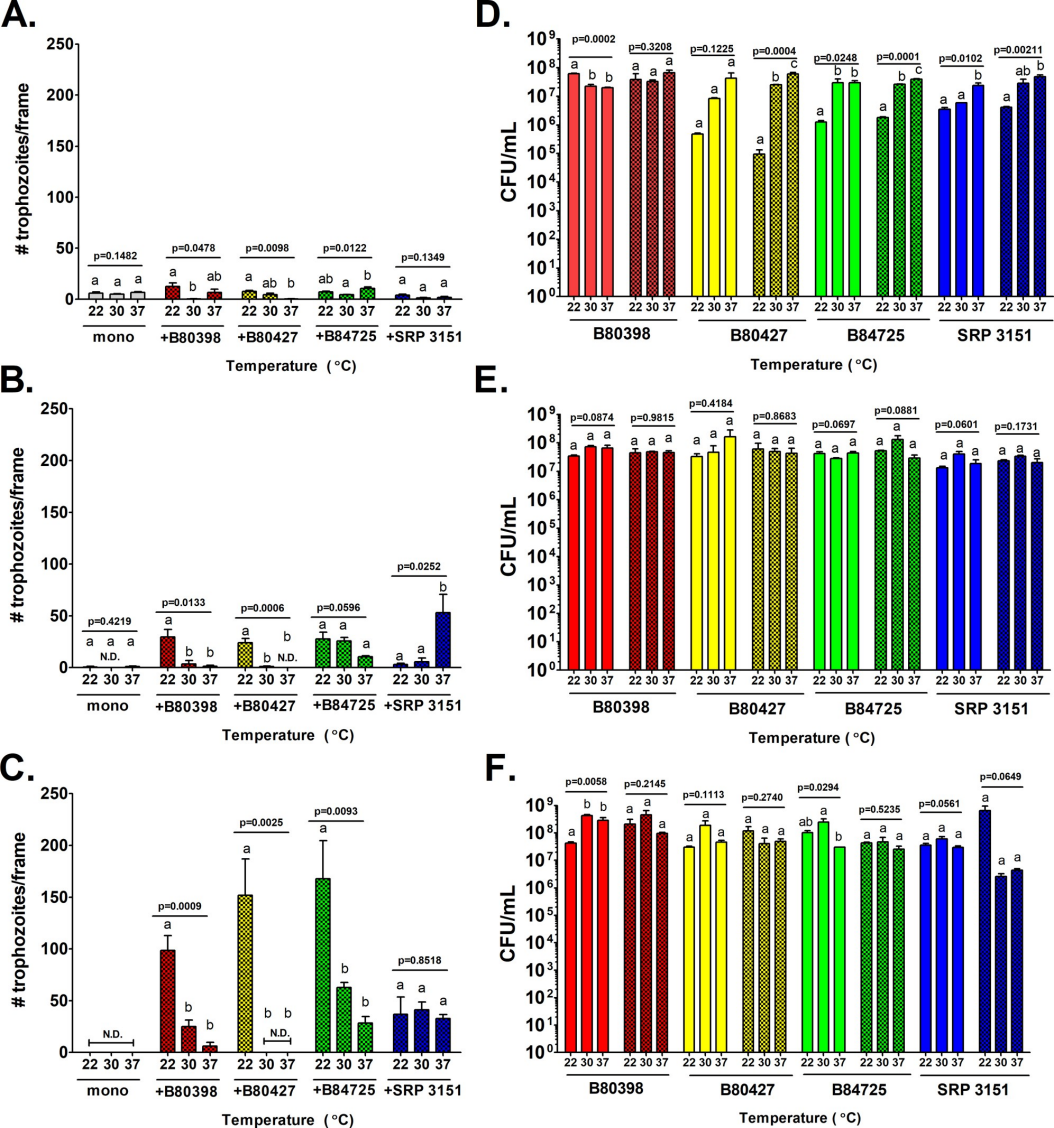
22°C supports *A. castellanii* co-cultured with *P*. *aeruginosa* over long periods of time. *A. castellanii* was cultured in monoculture and in co-culture with 4 *P*. *aeruginosa* strains in 1% HL5 at 22°C, 30°C, or 37°C. *A. castellanii* trophozoites and *P*. *aeruginosa* were enumerated via direct cell count at 400X magnification at (A and D) 1 day, (B and E) 7 days, and (C and F) 14 days. Panels A, B, and C represent *A. castellanii* trophozoites levels while panels D, E, and F represent *P*. *aeruginosa* survival. Data were analyzed with one-way ANOVA with Tukey’s post-test (n = 3 biological replicates). Lowercase letters indicate individual comparisons within each strain (but not between strains) and overall p-values for each group are indicated for each independent group comparison. N.D. indicates values beneath the limit of detection.

*A. castellanii* was cultured in monoculture and in co-culture with *P*. *aeruginosa* for 14 days in 1% HL5 at 22°C, 30°C, and 37°C. Cultures were examined at three time points: 1-, 7-, and 14-days post-inoculation. For *A. castellanii*, the number of trophozoites in monoculture was small but detectable at day 1 but virtually all cells had encysted or died 7- and 14-days post-inoculation (Figs 3A–3C and S5). This was expected as 1% HL5 is nutrient poor and does not support metabolically active trophozoites resulting in the conversion to metabolically dormant cysts in monoculture. In fact, cyst levels were generally higher in co-culture compared to mono-culture in higher temperatures, particular at later time points (S5 Fig). In co-culture, there were usually more *A. castellanii* trophozoites present in the 22°C condition than were present in the 30°C or 37°C conditions at all three time points (Fig 3A–3C). One exception to this is the high abundance of *A. castellanii* observed when co-cultured with SRP 3151 at day 7 at 37°C (Fig 3B). However, because this trend was not observed at day 14, this is likely due to biological variability.

In general, *P*. *aeruginosa* grew to higher concentrations at 37°C compared to 30°C or 22°C as expected, though growth was robust 7- and 14-days post- inoculation at all temperatures for all strains (Fig 3D–3F). This could be due to the exhaustion of all available nutrients in the media (except for dead cell turnover) after a few days. Taken together, these results indicate that 22°C is a suitable temperature to maintain the survival of *P*. *aeruginosa* with the vegetative form of *A. castellanii* in co-culture over a long period of time.

### The influence of inoculation ratios on co-culture dynamics

Since *A. castellanii* is an avid predator of *P*. *aeruginosa*, it is possible that a high amoebae:bacterium inoculation ratio will result in the eradication of the bacteria. It has also been previously shown that at low amoebae:bacterium ratios, *P*. *aeruginosa* rapidly kills *A. castellanii* [37]. Therefore, we asked whether there is a middle ground that allows for the survival of both partners. We hypothesized that a starting concentration of fewer *P*. *aeruginosa* compared to *A. castellanii* would result in higher *A. castellanii* trophozoite viability in co-culture long term while not having a large impact on *P*. *aeruginosa* survival as *P*. *aeruginosa* has a much shorter doubling time than the amoebae.

To test this hypothesis, *A. castellanii* and *P*. *aeruginosa* were co-cultured with different starting ratios of *A. castellanii* to *P*. *aeruginosa* to identify which inoculation ratio resulted in the highest viability of *A. castellanii* trophozoites and *P*. *aeruginosa* cells over time. The cells were combined in ratios of 100:1, 10:1, 1:1, 1:10, or 1:100 amoebae:bacteria. Monocultures of *A. castellanii* and *P*. *aeruginosa* were not assessed in this experiment as they would not further our understanding of co-culture dynamics; furthermore, we have already established the growth patterns of each of these species in 1% HL5 at room temperature in the previous experiments mentioned in this study. In addition, because *P*. *aeruginosa* exhibits robust growth in the abiotic conditions described for this experiment, *P*. *aeruginosa* cells were not quantitated in this experiment.

**Fig 4:**
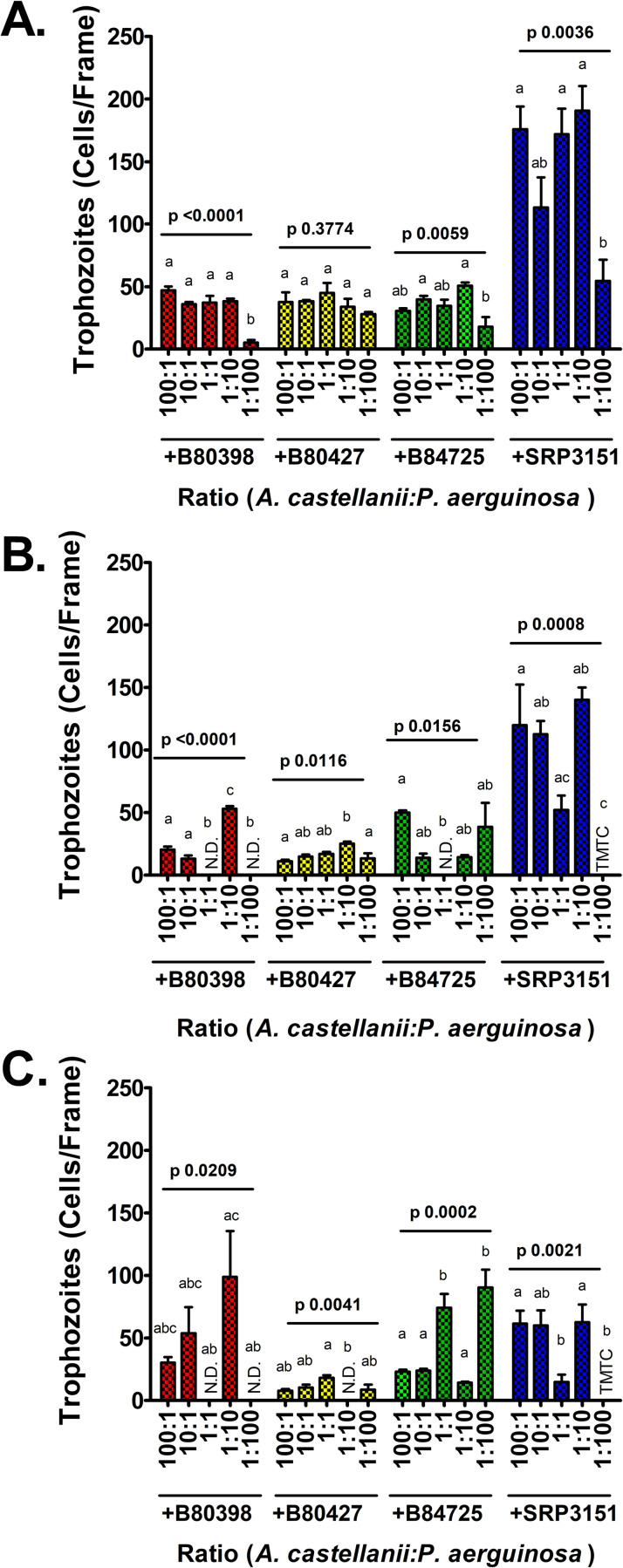
Higher initial doses of *A. castellanii* to *P*. *aeruginosa* cells resulted in consistent survival of both partners overall. *A. castellanii* (AC) was cultured in co-culture with 4 *P*. *aeruginosa* (PA) strains at ratios of 100 AC to 1 PA, 10 AC to 1 PA, 1 AC to 1 PA, 1 AC to 10 PA, and 1 AC to 100 PA in 1% HL5 at 22°C for 14 days. *A. castellanii* trophozoites were enumerated via direct cell count at 400X magnification at (A) 1 day, (B) 7 days, and (C) 14 days. Data analyzed with one-way ANOVA with Tukey’s post-test (n = 3 biological replicates). Lowercase letters indicate individual comparisons within each strain (but not between strains) and overall p-values for each group are indicated for each independent group comparison. N.D. (not detected) indicates values less than the lower limit of detection. TMTC (too many to count) indicates levels higher than the upper limit of detection.

The different ratios of cells were incubated in 1% HL5 at 22°C (based on our previous results) and enumerated at 1, 7, and 14 days. We observed that amoebae survival and proliferation in co-culture at these different ratios varied strongly depending on *P*. *aeruginosa* strain. On days 1 and 7, *P*. *aeruginosa* SRP 3151 allowed for ∼3 times the levels of amoebae retention compared to the other *P*. *aeruginosa* strains on days 1 and 7. This effect was not observed on day 14 when survival of co-cultures *A. castellanii* with *P*. *aeruginosa* SRP 3151 was much lower in most conditions and *P*. *aeruginosa strains* B80398, B84725, and SRP 3151 all had similar levels of trophozoites (Fig 4). In contrast, *P*. *aeruginosa* B80427, the small colony variant, supported the least amount of trophozoites at day 14 overall. In general, the ratios of 1:1 and 1:10 amoeba:bacteria tended to support the highest trophozoite levels across the time points though there was a great deal of variability in these data, and it may just be dependent on the strain used. However, the 1:100, 1:10, and 1:1 ratios resulted in reductions of trophozoites to levels below the limit of detection on days 7 and/or 14 for some strains. This could be inhibitory for long term studies and may result in bottlenecks in the population. Therefore, the ratio of amoebae to bacteria that supported the most consistent survival of both partners was 100:1 or 10:1 amoeba:bacterium for the strains tested.

Cyst formation was high for all starting ratios after 1 day of incubation and mostly remained steady over 7 and 14 days (S5 Fig). Notable exceptions include a very high level of cysts when *A. castellanii* was co-cultured with SRP 3151, the environmental isolate, at a starting ratio of 1 amoeba:100 bacteria after 1 day and levels over the limit of detection after 7- and 14-days post-inoculation. Taken together, these results suggest that ratios with levels of *A. castellanii* equal or greater to the levels of *P*. *aeruginosa* will most consistently result in the retention of *A. castellanii* and *P*. *aeruginosa* together over the long term.

## Discussion

*A. castellanii* and *P*. *aeruginosa* can be cultured together with high viability of both partners if the correct abiotic conditions are utilized. Low nutrient culture medium (1% HL5) is imperative to the co-culture survival of both organisms as high concentrations of nutrients resulted in the death of *A. castellanii* in co-culture with *P*. *aeruginosa*. Similarly, temperature is also an important abiotic factor as 22°C resulted in the most robust survival of *A. castellanii* when co-cultured with multiple *P*. *aeruginosa* strains while 37°C resulted in a significant reduction in viable *A. castellanii* cells in co-culture. Biotic factors such as life history of *P*. *aeruginosa* and the initial inoculum dose of each organism proved to have strain-dependent impacts on *A. castellanii* survival in co-culture. We concluded from these studies that the conditions that ensured the robust survival of both amoebae and multiple bacterial strains were growth in 1% HL5 medium, incubation at 22°C, and a starting inoculum ratio of 10 to 100 amoebae: 1 bacterium. We note here, however, that we did not recursively test all these conditions for all strains. Therefore, there could be more “optimal” conditions, depending on the strain itself. Further, we only tested a single strain of amoeba and other strains of *A. castellanii* may have different co-culture dynamics with *P*. *aeruginosa* or other metabolic differences compared to the strain used in this study.

Our results with respect to nutrient requirements are consistent with other bacteria-*A. castellanii* co-culture systems and demonstrate that *A. castellanii* is better equipped to tolerate co-culture with many bacterial species, including *P*. *aeruginosa*, under low nutrient conditions compared to high nutrient conditions as well as below a 100:1 bacteria to amoebae ratio [37]. Our experiments revealed that 22°C is a good co-culture temperature for these two species while 37°C resulted in high morbidity for *A. castellanii* when co-cultured with most of our *P*. *aeruginosa* strains tested. This result is similar to previously published literature that described experiments between *A. castellanii* and *Yersinia enterocolitica* where *A. castellanii* viability was reduced in both monoculture and co-culture at 37°C but both *Y*. *enterocolitica* and *A. castellanii* remained viable in the lower temperatures tested (7°C and 25°C) [63]. Similar results were also observed between *A. castellanii* and *Campylobacter jejuni* where *A. castellanii* viability was inhibited by the *C*. *jejuni* at 37°C but not at 25°C [64].

Increased survival of *A. castellanii* co-cultured with *P*. *aeruginosa* in lower-nutrient culture media and at temperatures below 37°C could be due to the fact that expression of virulence factors by *P*. *aeruginosa* is metabolically costly and some virulence factors have been shown to only be expressed at 37°C in clinical isolates [61, 65]. Though it could be argued that long-term experimental evolution systems utilizing *P*. *aeruginosa* should be conducted at 37°C because it is the temperature at which *P*. *aeruginosa* is able to express many of its virulence factors and is also the temperature these organisms would experience inside a human host. It can also be argued that 22°C is the temperature these organisms would experience in their natural, non-mammalian habitat and thus would be the better temperature to assess co-culture survival and co-evolution between them.

Life history and colony phenotype did not have a substantial impact on *A. castellanii* survival in co-culture at an initial dose of 1 amoeba to 1 bacterial cell; however, there is a marked impact on *A. castellanii* survival when the initial dose of amoebae to bacteria is altered. *P*. *aeruginosa* strains from clinical origin proved to be more deadly overall to the amoebae than the environmental isolate tested by 14-days of co-culture in most of the ratios tested. Interestingly, this result is opposite to results observed when *A. castellanii* is co-cultured with clinical and environmental strains of *L*. *pneumophila*, though the only dose tested in that study was 1 amoebae to 10 bacteria cells [66]. Our results could be due to the fact that some of these clinical isolates could have been recently acquired by the host and thus their life history would be more liken to the environmental isolates tested; however, this is unlikely, particularly with respect to the small colony and mucoid isolates as these phenotypes (*P*. *aeruginosa* B84725 and B80427 respectively) are associated with chronic infection and heretofore are rarely if at all isolated from non-host-associated systems. Conversely, it is also possible that the environmental isolates could have recently been excreted from a human host into the bathroom/kitchen sink drains from which they were isolated, and we have no means to rule out this possibility.

The results of these experiments give insights into the complicated dynamics of polymicrobial communities when multiple species can act as predators of other community members, which is a common trope in the microbial world but less so in the animal kingdom where the trophic levels are more defined. Researchers can also use this information to look further into these specific interactions. For example, with the co-culture variables identified, it is possible to increase the duration of co-culture of *A. castellanii* and *P*. *aeruginosa* without needing to replenish the amoebae or bacteria for long-term experimental evolution experiments as seen in the research conducted by Leong et al. [67]. This might be useful for studies that seek to test broad fundamental evolutionary or ecological hypotheses in addition to further understanding other polymicrobial, interkingdom interactions.

## Supporting information

S1 Fig *P*. *aeruginosa* viable cell count in monoculture and in co-culture with *A. castellanii* in PBS for 1-, 3-, 5-, or 7-days. Viable cell counts of *P*. *aeruginosa* strains are provided for monoculture (solid bars) and co-culture with *A. castellanii* (patterned bars). Each *P*. *aeruginosa* strain is shown in its own panel with (A) B80398, (B) B80427, (C) B84725, (D) B80422, (E) PA B80425, (F) PA B84723, (G) PA3, (H) SRP3151, (I) SRP 17–047, and (J) SRP 17–055. Data analyzed with t-tests comparing monoculture to co-culture at each time point (asterisks directly above bars) or with one-way ANOVAs for relevant grouped data (bracketed asterisks)Asterisks indicate p-values (* p<0.05, ** p<0.01, *** p<0.001, **** p<0.0001).
(TIF)

S2 Fig *Acanthamoeba castellanii* cell count in monoculture and in co-culture with *Pseudomonas aeruginosa* in PBS for 7-days. Direct cell counts of *A. castellanii* (A) trophozoites and (B) cysts in monoculture and in co-culture with *P*. *aeruginosa* are provided. Data analyzed with 1-way ANOVA with Dunnett’s post-test comparing each co-culture condition to monoculture at each time point.
(TIF)

S3 Fig *A. castellanii* survival in co-culture with clinical versus environmental *P*. *aeruginosa* strains. The mean survival of *A. castellanii* trophozoites cultured with the 7 clinical isolates or 3 environmental isolates grouped data are shown. Data analyzed with t-test between groups at each time point. There were no significant differences between the two groups at any time point.
(TIF)

S4 Fig Growth patterns of *A. castellanii* in mono-culture in different levels of HL5 medium. Trophozoites (A, C) or cysts (B, D) were enumerated over the course of 6 days. Panels A and B show data when comparing *A. castellanii* incubated in 1X PBS (no carbon sources in theory) to 100% HL5 (rich medium). Panels C and D show data collected from *A. castellannii* cultured in a 10-fold dilution of HL5 to further define its growth dynamics over the course of 6 days. Triplicate biological replicates were quantitated by microscopy from multiple images. Error bars represent the standard deviation of the mean.
(TIF)

S5 Fig Cyst concentration in mono and co-culture based on (A-C) nutrient concentration, (D-F) temperature, or (G-I) amoeba:bacteria starting ratio. Legends for each condition are shown only on the rightmost panel. Panels A, D, and G represent data at 1 day after inoculation, panels B, E, and H represent data after 7 days post-inoculation, and panels C, F, and I represent data after 14 days post-inoculation. Error bars represent standard deviation of the mean. N.D. indicates cyst levels were not above the limit of detection. TMTC indicates too many to count in a frame.
(TIF)

S1 Table Two-way ANOVA on the effects of *P*. *aeruginosa* strain and time on the survival of *P*. *aeruginosa* and *A. castellanii* in PBS. Table summarizes the 2-way ANOVA analysis output for Fig 1. *DF (degrees of freedom), SS (summary of squares), MS (mean of squares).
(DOCX)
