# Supplementary figures and images for: Examining the influence of environmental factors on Acanthamoeba castellanii and Pseudomonas aeruginosa in co-culture
Source: PLoS One. 2024 Jun 24;19(6):e0305973. doi: 10.1371/journal.pone.0305973 (PMC11195979; doi:10.1371/journal.pone.0305973)

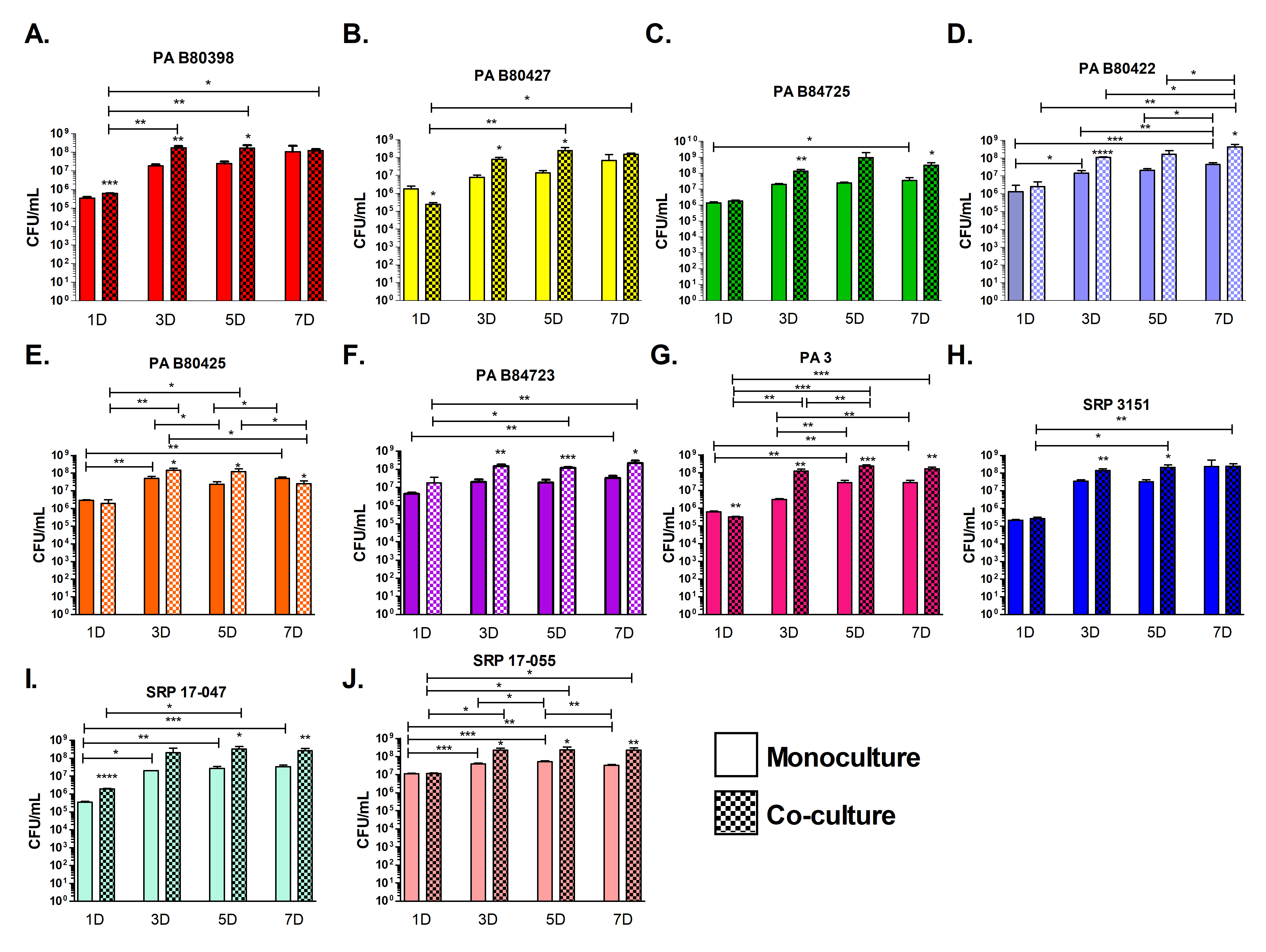

Supplement: S1 Fig — Viable cell counts of P. aeruginosa strains are provided for monoculture (solid bars) and co-culture with A. castellanii (patterned bars). Each P. aeruginosa strain is shown in its own panel with (A) B80398, (B) B80427, (C) B84725, (D) B80422, (E) PA B80425, (F) PA B84723, (G) PA3, (H) SRP3151, (I) SRP 17–047, and (J) SRP 17–055. Data analyzed with t-tests comparing monoculture to co-culture at each time point (asterisks directly above bars) or with one-way ANOVAs for relevant grouped data (bracketed asterisks)Asterisks indicate p-values (* p<0.05, ** p<0.01, *** p<0.001, **** p<0.0001). (TIF) [file pone.0305973.s001.tif]

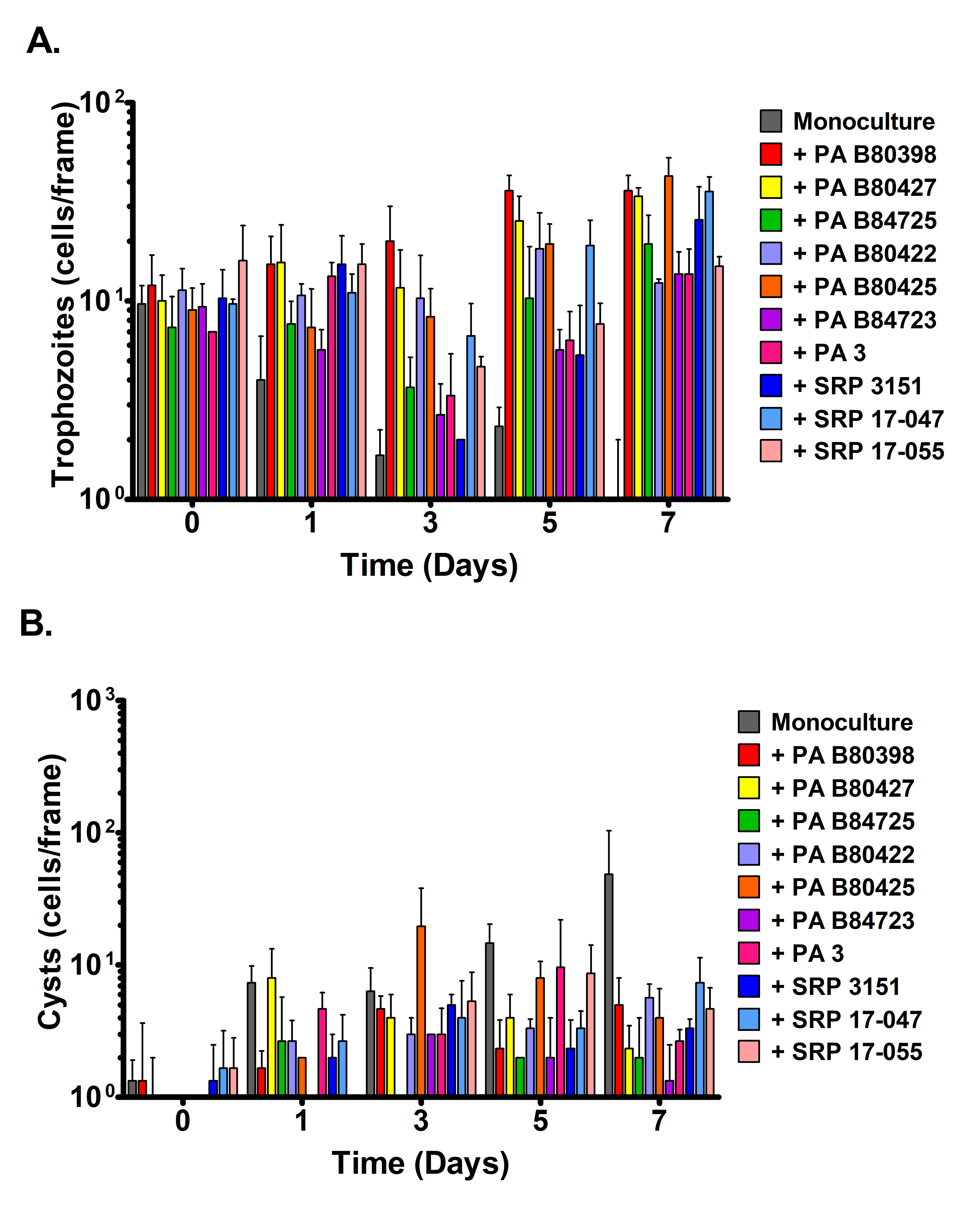

Supplement: S2 Fig — Direct cell counts of A. castellanii (A) trophozoites and (B) cysts in monoculture and in co-culture with P. aeruginosa are provided. Data analyzed with 1-way ANOVA with Dunnett’s post-test comparing each co-culture condition to monoculture at each time point. (TIF) [file pone.0305973.s002.tif]

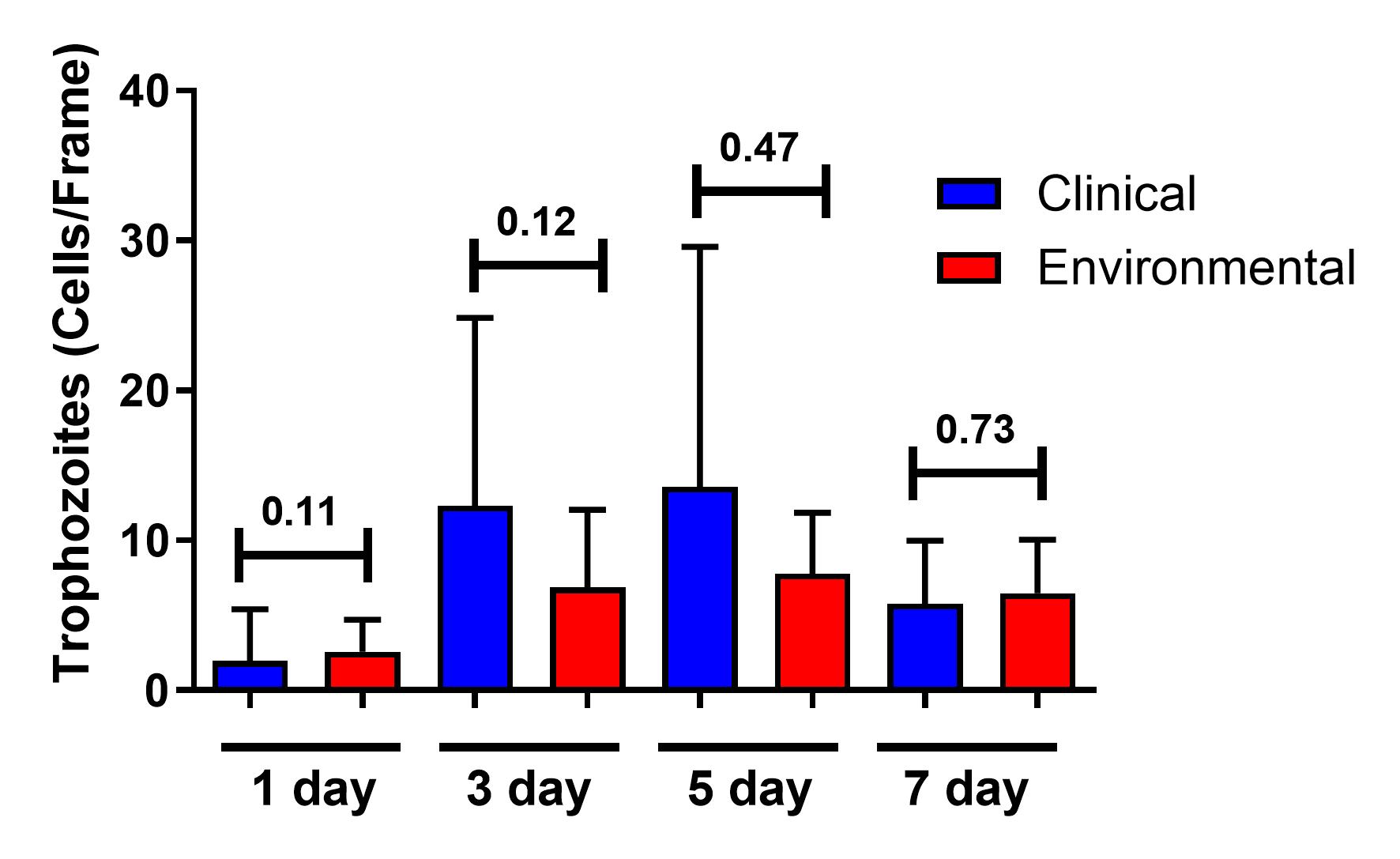

Supplement: S3 Fig — The mean survival of A. castellanii trophozoites cultured with the 7 clinical isolates or 3 environmental isolates grouped data are shown. Data analyzed with t-test between groups at each time point. There were no significant differences between the two groups at any time point. (TIF) [file pone.0305973.s003.tif]

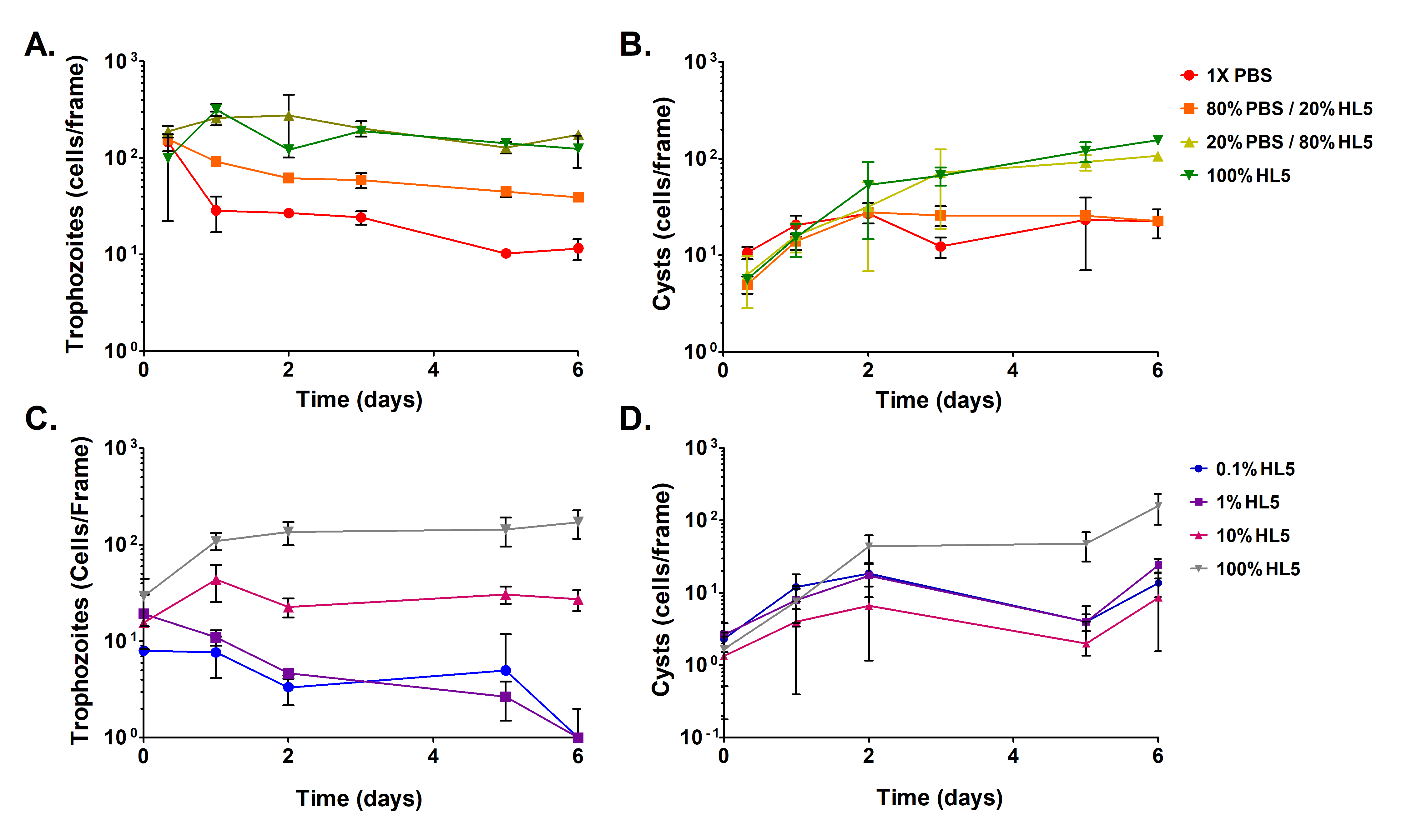

Supplement: S4 Fig — Trophozoites (A, C) or cysts (B, D) were enumerated over the course of 6 days. Panels A and B show data when comparing A. castellanii incubated in 1X PBS (no carbon sources in theory) to 100% HL5 (rich medium). Panels C and D show data collected from A. castellannii cultured in a 10-fold dilution of HL5 to further define its growth dynamics over the course of 6 days. Triplicate biological replicates were quantitated by microscopy from multiple images. Error bars represent the standard deviation of the mean. (TIF) [file pone.0305973.s004.tif]

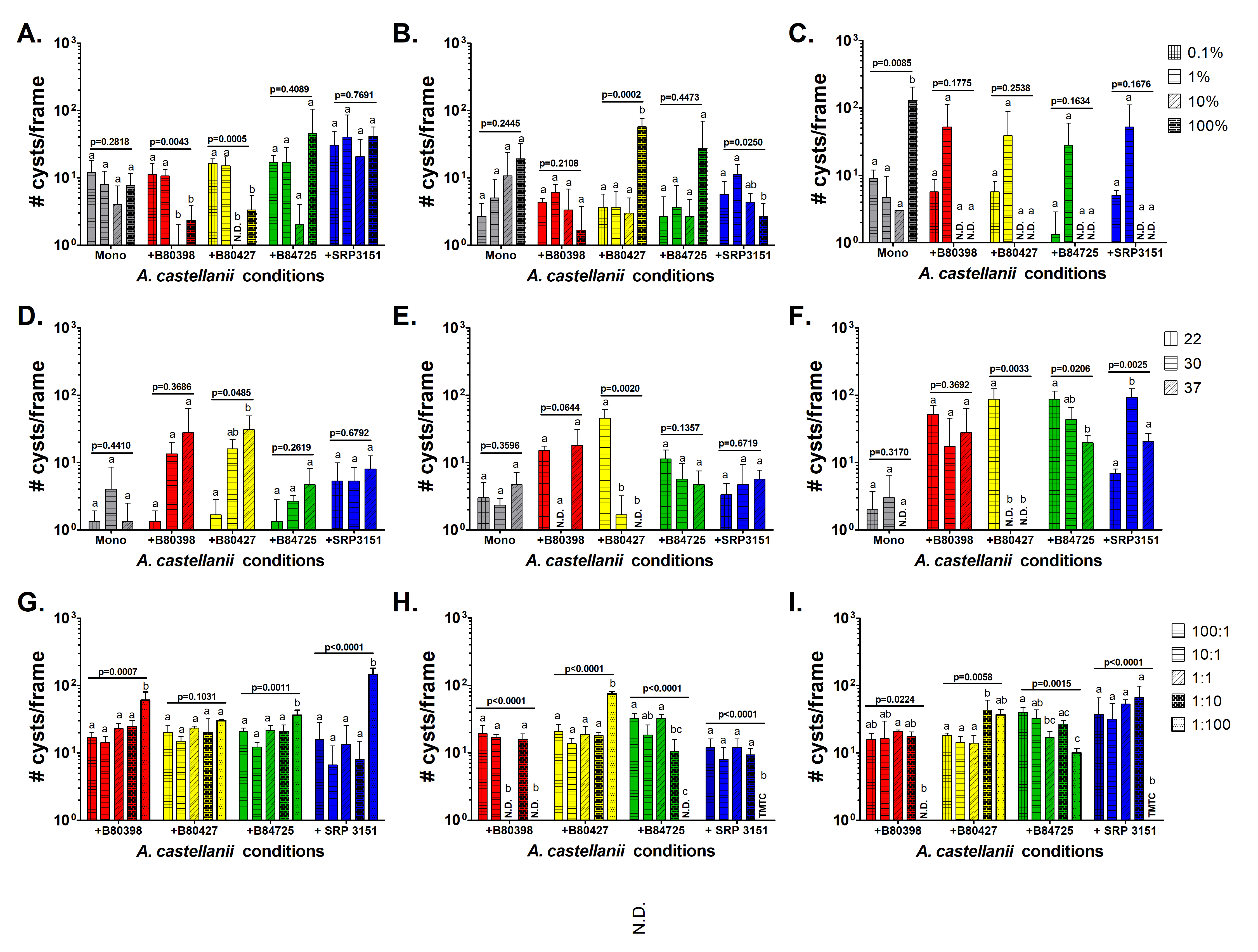

Supplement: S5 Fig — Cyst concentration in mono and co-culture based on (A-C) nutrient concentration, (D-F) temperature, or (G-I) amoeba:bacteria starting ratio. Legends for each condition are shown only on the rightmost panel. Panels A, D, and G represent data at 1 day after inoculation, panels B, E, and H represent data after 7 days post-inoculation, and panels C, F, and I represent data after 14 days post-inoculation. Error bars represent standard deviation of the mean. N.D. indicates cyst levels were not above the limit of detection. TMTC indicates too many to count in a frame. (TIF) [file pone.0305973.s005.tif]
